# High Frequency of Microvascular Dysfunction in US Outpatient Clinics: A Sign of High Residual Risk? Data from 7,105 Patients

**DOI:** 10.1155/2022/4224975

**Published:** 2022-01-06

**Authors:** Morteza Naghavi, Stanley Kleis, Hirofumi Tanaka, Albert A. Yen, Ruoyu Zhuang, Ahmed Gul, Yasamin Naghavi, Ralph Metcalfe

**Affiliations:** ^1^SHAPE, Palo Alto, CA, USA; ^2^American Heart Technologies, LLC, USA; ^3^University of Houston, Houston, TX, USA; ^4^The University of Texas at Austin, Austin, TX, USA; ^5^Endothelix, Inc., Houston, TX, USA

## Abstract

Previous studies have linked peripheral microvascular dysfunction measured by arterial tonometry to high residual risk in on-statin patients. Digital thermal monitoring (DTM) of microvascular function is a new and simplified technique based on fingertip temperature measurements that has been correlated with the burden of atherosclerosis and its risk factors. Here, we report analyses of DTM data from two large US registries: Registry-I (6,084 cases) and Registry-II (1,021 cases) across 49 US outpatient clinics. DTM tests were performed using a VENDYS device during a 5-minute arm-cuff reactive hyperemia. Fingertip temperature falls during cuff inflation and rebounds after deflation. Adjusted maximum temperature rebound was reported as vascular reactivity index (VRI). VRI distributions were similar in both registries, with mean ± SD of 1.58 ± 0.53 in Registry-I and 1.52 ± 0.43 in Registry-II. In the combined dataset, only 18% had optimal VRI (≥2.0) and 82% were either poor (<1.0) or intermediate (1.0-2.0). Women had slightly higher VRI than men (1.62 ± 0.56 vs. 1.54 ± 0.47, *p* < 0.001). VRI was inversely but mildly correlated with age (*r* = −0.19, *p* < 0.001). Suboptimal VRI was found in 72% of patients <50 years, 82% of 50-70 years, and 86% of ≥70 years. Blood pressure was not correlated with VRI. In this largest registry of peripheral microvascular function measurements, suboptimal scores were highly frequent among on-treatment patients, possibly suggesting a significant residual risk. Prospective studies are warranted to validate microvascular dysfunction as an indicator of residual risk.

## 1. Introduction

Of the three layers of an artery, the intima or endothelial layer has gathered the most attention as it is a critical regulator of the overall hemodynamic function [1–3]. It is involved in controlling vascular homeostasis and repair and regulating blood pressure and blood flow acting via vascular tone. In response to the increased shear stress, the endothelium produces nitric oxide and other vasodilating substances causing the arterial wall to dilate. A healthy endothelium is highly reactive to such stimuli and causes blood flow to increase markedly and promptly [1–3]. Reactive hyperemia that produces shear stress is the most practical and commonly used way of assessing endothelial function. This function can be measured both at macrovascular (conduit artery) and microvascular levels.

**Figure 1:**
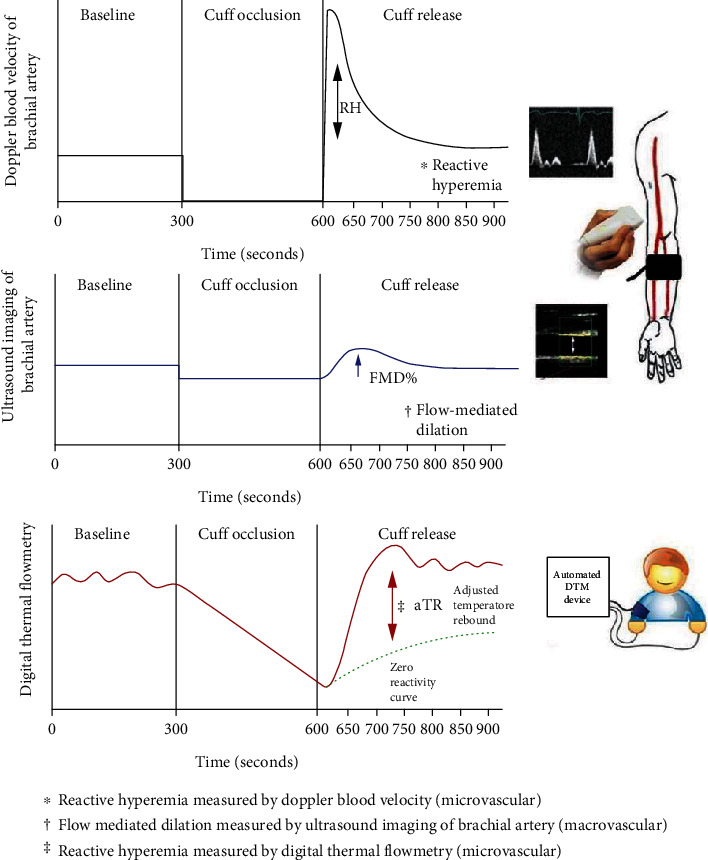
Comparisons between reactive hyperemia (RH) and flow-mediated dilation (FMD) measured by ultrasound imaging versus digital thermal monitoring (DTM).

Flow-mediated dilation (FMD) evaluates endothelial function at the conduit (brachial) artery level [4, 5], whereas methodologies that focus on the microvascular level including peripheral arterial tonometry (PAT) and the presented data on microvascular (endothelial) dysfunction have been made available [3, 6]. In both methods, the endothelial function measurement is performed following a brief period of interrupting blood flow causing ischemia. We and others have demonstrated that digital thermal monitoring (DTM) of microvascular function is a simplified, noninvasive method that is much easier, more feasible, and practical for clinical settings than ultrasound imaging involved in FMD (Figure 1). These data demonstrate that DTM correlates well with the presence of atherosclerotic cardiovascular disease and its risk factors [7–12]. Herein, we present analyses of two DTM registries comprising a total of 7,105 tests performed in 49 outpatient clinics across the Unites States. The working hypothesis was that a very high level of suboptimal vascular dysfunction exists across sex/gender and age groups.

## 2. Methods

**Figure 2:**
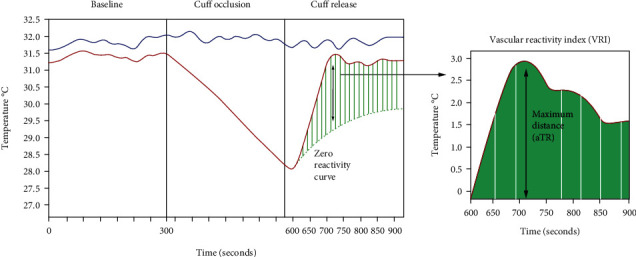
Sample test for digital thermal monitoring (DTM) of vascular reactivity. A sample screen displays the right finger temperature curve (red), the left finger temperature curve (blue), and the zero reactivity curve (green). The vascular reactivity index (VRI) is taken as the adjusted maximum value of the temperature curve during the reactive hyperemic period. Zero reactivity curve (ZRC) is the green line, calculated based on predicted temperature rebound in the right finger if no reactive hyperemia were elicited by the 5-minute cuff occlusion.

The procedure for measuring microvascular function using DTM has been described previously in detail [5, 13–16]. All DTM tests were performed using a VENDYS device (Endothelix, Palo Alto, CA), a nonimaging, simplified system that fully automates the arm-cuff induced reactive hyperemia protocol and measurements. A schematic VRI test report is shown in Figure 2. In preparation, a blood pressure cuff was placed on the right upper arm, and skin temperature sensors were affixed to both index fingers. The software-controlled DTM test began with an automated measurement of blood pressure and heart rate obtained from the arm cuff. Following a 5-minute baseline period of temperature stabilization, a 5-minute cuff occlusion (cuff inflated to 50 mmHg above systolic blood pressure) of the right arm was performed. During the cuff occlusion period, fingertip temperature in the right hand decreased because of the absence of warm circulating blood. When the cuff was released after the 5-minute occlusion, blood flow to the forearm and hand was restored, and this resulted in a “temperature rebound” in the fingertip that was directly related to the hyperemic blood flow response resulting from microvascular reactivity [14]. Using the recorded fingertip temperatures, the ambient temperature of the testing room, the observed slope of temperature decline during cuff occlusion, and a multivariate bioheat formula, the VENDYS software calculated and plotted a zero reactivity curve (ZRC). The ZRC served as an internal control and showed the expected temperature rebound curve, if zero vascular reactivity was present and the other variables remained the same. In other words, the ZRC was the expected temperature curve, if no vasodilation and subsequent reactive hyperemia had occurred [13]. Vascular reactivity index (VRI) was determined by first taking the maximum difference between the observed temperature rebound curve and the ZRC during the reactive hyperemia period and then adjusting it for starting fingertip temperature and ambient temperature. VRI ranges from 0.0 to 3.5 and is classified as being indicative of poor (0.0 to <1.0), intermediate (1.0 to <2.0), or good (≥2.0) microvascular function. Very few individuals exhibit VRI equal to or greater than 3.0 which correlates to an excellent vascular reactivity index [5, 13–17].

The DTM test registries included age, sex/gender, blood pressure, heart rate, and fingertip temperature recorded during DTM tests. The registries did not include other health-related information. All DTM tests were performed in outpatient clinical settings. The first VENDYS registry included 6,084 patients tested between 2011 and 2016, here referred to as Registry-I. The second registry included 1,021 patients tested between 2017 and the first half of 2018, here referred to as Registry-II. Overall, this study included a total of 7,105 patients. Data for both registries were collected from 49 outpatient clinics in the US.

Statistical analyses were performed using RStudio (RStudio: Integrated Development for R. RStudio, Boston, MA) and MATLAB (The MathWorks, Natick, MA). Descriptive statistics were expressed as the mean ± SD and categorical variables as percentages. VRI scores in men and women were compared using unpaired Student's *t*-test. Comparisons of categorical data (e.g., proportion of subjects with good VRI in men vs. women) were performed using Fisher's exact test. Pairwise correlations were performed using Pearson's correlation and regression analyses. Associations between VRI and multiple patient characteristics (e.g., age, sex/gender, blood pressure, and heart rate) were evaluated using bidirectional (forward and backward) multiple stepwise regression analyses. A *p* value of <0.05 was considered statistically significant. When performing statistical comparisons, tests with missing data were excluded from the comparison.

## 3. Results

Basic characteristics of patients and DTM tests for Registry-I and II are shown in Table 1. Overall, the study populations in both registries were similar in terms of age and sex/gender and were representative of patient population seen in internal medicine and cardiology outpatient clinics in the US. Key characteristics included age 65 ± 12 years in Registry-I and 60 ± 13 in Registry-II. Systolic blood pressure was 137 ± 20 mmHg in Registry-I and 129 ± 19 mmHg in Registry-II.

**Figure 3:**
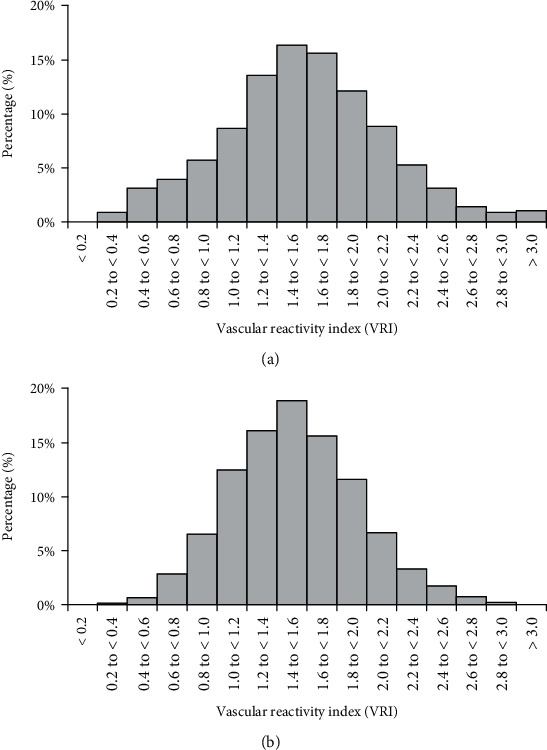
Distributions of vascular reactivity index (VRI) in Registry-I (a) and in Registry-II (b).

**Figure 4:**
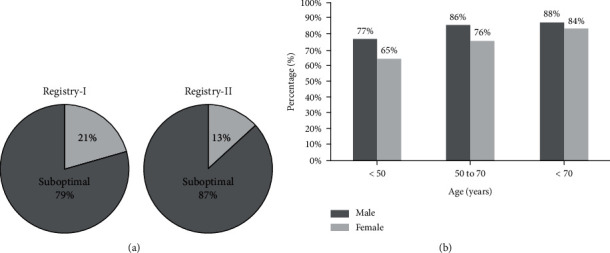
Prevalence of optimal and suboptimal vascular reactivity index (VRI) in Registry-I and Registry-II (a) and by gender and age group (b).

The VRI distributions in both registries are shown in Figure 3. 79% of patients in Registry-I and 87% in Registry-II were categorized as having suboptimal VRI (Figure 4). Among the suboptimal cases, 66% of Registry-I and 77% of Registry-II were classified as intermediate and 13% of Registry-I and 10% of Registry-II were categorized as poor. Average VRI in Registry-I was slightly higher in women than men (1.63 ± 0.60 vs. 1.53 ± 0.50, *p* < 0.001), whereas VRI was nearly identical in men (1.51 ± 0.43) and women (1.52 ± 0.43) in Registry-II (Table 1).

**Figure 5:**
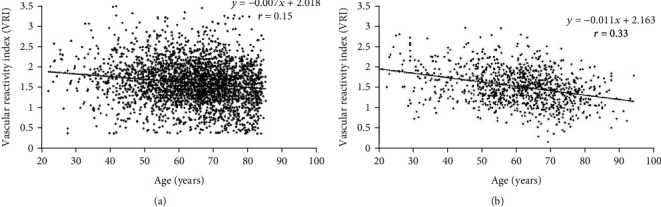
Associations between vascular reactivity index (VRI) and age in Registry-I (a) and in Registry-II (b).

The distribution of poor, intermediate, and good VRI in both registries is shown in Figure 3. The percentage of good VRI was greater in women than in men (23.7 vs. 15.2%, *p* < 0.001). However, prevalence of poor VRI was not different between men and women (12.3 vs. 13.3%, *p* = 0.4). VRI was inversely and significantly correlated with age (*r* = −0.19, *p* < 0.001) (Figure 5). Poor VRI (<1.0) was most frequent in the oldest age group (>70 yrs., 19.7%) compared with middle age (50–70 yrs., 9.3%) and younger (<50 yrs., 9.1%) (*p* < 0.001). However, the distribution of poor, intermediate, and good VRI values in the oldest age group was similar to that of the overall study population (20% poor, 66% intermediate, and 14% good). 77% of men under 50 and 92% of women under 50 were classified as suboptimal. 87% of men over the age 50 and 79% of women over the age 50 were classified as suboptimal. VRI showed no correlations with systolic and diastolic blood pressure, pulse pressure, or heart rate. This was true for both men and women. As shown in Table 2, multiple stepwise regression models were built using VRI as the dependent variable and age, sex/gender, systolic and diastolic blood pressure, and heart rate as independent variables. Only age, sex/gender, and heart rate remained in the model as significant predictors of VRI, but the overall model only accounted for 5.4% of total variability of VRI.

## 4. Discussion

To our knowledge, this is the largest database to date on endothelial function measurement including 7,105 cases. The second largest is the data from the Framingham Heart Study with 7,031 FMD measurements [18]. The most important observation of the present study is a very high level of suboptimal VRI that is consistent across sex/gender and age groups. Although the Framingham Heart Study and other community-based investigations have reported endothelial dysfunction in apparently healthy adults [18–22], such a high frequency in patients under therapy is concerning and raises questions regarding “residual risk.” Our data show that a large number of patients who are receiving guideline-based treatments in medical clinics across the USA continue to exhibit a suboptimal VRI suggestive of persistent endothelial dysfunction. If corroborated by other registries, these findings should be taken as a “wake-up call” for physicians to pay much more attention to residual risk in patients already under treatment.

**Figure 6:**
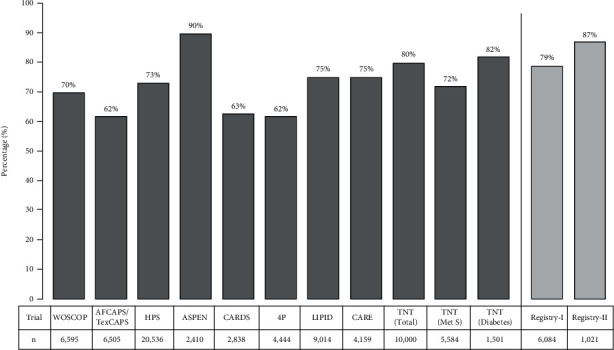
A hypothetical comparison of high residual risk reported by major statin trials versus the high sub-optimal vascular reactivity index (VRI) in digital thermal monitoring (DTM) registries. Created based on “Residual risk: Is LDL target enough? [45].

Reports of high residual risk in on-statin patients are well documented in the literature [23–28]. Among the potential determinants of residual risk, abnormal lipoprotein particles have received the most attention [24, 29, 30]. Recent efforts, including the REDUCE-IT trial targeting lipoprotein (a) for lowering residual risk, have been promising [31, 32]. However, a variety of other factors, such as smoking, emotional stress, inflammatory disorders, and poor oral hygiene, may not be adequately addressed and may contribute to the hidden residual risk. Accordingly, monitoring endothelial function has emerged as a promising candidate [33]. Indeed, measurement of microvascular endothelial function predicts risk in coronary artery disease patients [34]. Because patients' LDL levels were well controlled in their study, the authors concluded that persistent microvascular endothelial dysfunction reflected residual risk [34]. Similarly, microvascular dysfunction has been used to detect the residual risk and predict outcomes in patients with coronary artery disease who were treated successfully with statin therapy [35]. Others have hinted at a similar role for endothelial dysfunction in diabetic patients [36, 37]. Persistent impairment of endothelial vasomotor function despite an optimized risk factor therapy predicted poor outcomes in coronary artery disease patients [38]. As one of the inflammatory factors, hsCRP has been proposed as a reliable marker of residual risk. However, this view is not largely shared mainly due to poor specificity of hsCRP to arterial wall and atherosclerosis [39–44]. To date, there is insufficient data to determine reasonable markers of residual risk. As shown in Figure 6, the residual risk bars in those major statin trials look similar to the suboptimal VRI seen in the present registries [45].

Another interesting observation from the present study is the lack of correlations between endothelial function measured by DTM technique and arterial blood pressure. This finding is not unique to DTM. Studies with FMD and peripheral arterial tonometry (PAT) have shown similar findings [46, 47]. The results of multivariable analyses showed that blood pressure had minimal correlations with age. Because blood pressure is known to correlate with age in untreated population [48], antihypertensive medications may have played a role in reducing the relationship. In view of other studies [46, 47], it is not surprising that blood pressure and VRI were not correlated because endothelial dysfunction can serve as a much earlier and more sensitive indicator of vascular abnormality than blood pressure. Despite a volatile nature, sustained hypertension is a more static condition due to gradual structural and functional adaptations of the arterial system. Indeed, when healthy volunteers were subjected to a single, high-calorie, high-fat meal, endothelial function measured by FMD immediately worsened postprandially whereas no changes in blood pressure were observed [50, 51]. Furthermore, endothelial dysfunction has been reported in children whereas hypertension is rarely found in pediatric populations [52, 53]. Moreover, endothelial function was a more reliable predictor of future hypertension than blood pressure itself and endothelial function predicted the transition among pre-hypertension patients who progressed to hypertension [54].

As reported in a community-based study of 5,000 individuals, traditional risk factors only accounted for 15% of FMD and 14% of PAT variability [56]. This may indicate that endothelial function provides a new angle into the status of vascular health. Although endothelial function of conduit arteries (macrovascular) measured by FMD was introduced as the noninvasive marker of endothelial function, there is no evidence regarding superiority of macrovascular over microvascular reactivity [55]. More studies are needed to evaluate the predictive value of each method for assessing cardiovascular risk and monitoring response to therapies. Functional measurements such as VRI offer a new window into an individual's vascular physiology at the time of measurement. They provide a direct assessment of arterial function. Structural markers such as coronary calcium and carotid artery IMT are good indicators of susceptibility to risk factors and show the effects of past exposure, but they do not show the current status or the activity level of the disease. Measurement of endothelial function and vascular reactivity provides a direct and instant assessment of the vascular physiology and is specific to the vessel wall.

The strengths of our study include a large sample size and a mixed population of men and women, geographically dispersed, and coming from various outpatient clinics throughout the USA. Therefore, the registries can provide a real-world assessment of the problem at large. The main limitation of the present study is that neither Registry-I nor Registry-II contained detailed clinical information about patients' diagnostic or therapeutic status. Therefore, we cannot be certain as to whether these patients were maximally treated and what percentage of them reached the target treatment goals. However, it is generally expected that basic standards of care according to existing guidelines are provided by clinics that utilize cutting-edge technologies such as endothelial function testing devices. Furthermore, the near-target average blood pressure levels may suggest that these patients were treated based on current standards of care. Given reports of high residual risk in other studies, it is unlikely that our results represent an anomaly [34, 35].

In conclusion, in this largest collection of peripheral microvascular function measurements in on-treatment patients, a high prevalence of suboptimal scores was found across both genders and all age groups. These findings suggest a possible high level of residual risk in this real-world data. More needs to be done to minimize residual risk, and further studies are warranted to validate the use of endothelial function testing, such as digital thermal monitoring, for detecting and monitoring residual risk.

## Figures and Tables

**Table 1:** Selected patient and test characteristics.

	Registry-I	Registry-II	Combined
	(*n* = 6,084)	(*n* = 1,021)	(*n* = 7,105)
Variable	Mean ± SD	Mean ± SD	Mean ± SD
Age (years)	65 ± 12	60 ± 13	63 ± 13
Men	63 ± 12	60 ± 13	62 ± 12
Women	66 ± 12	61 ± 14	65 ± 13
Men/women (%)	57/43	60/40	57/43
Vascular reactivity index (U)	1.58 ± 0.53	1.52 ± 0.43	1.57 ± 0.52
Men	1.53 ± 0.50	1.51 ± 0.43	1.54 ± 0.47
Women	1.63 ± 0.60	1.52 ± 0.43	1.62 ± 0.56
Systolic blood pressure (mmHg)	137 ± 20	129 ± 19	136 ± 20
Men	139 ± 20	130 ± 19	136 ± 20
Women	139 ± 22	128 ± 19	136 ± 22
Diastolic blood pressure (mmHg)	77 ± 12	72 ± 14	76 ± 13
Men	79 ± 11	74 ± 14	78 ± 13
Women	75 ± 12	68 ± 14	73 ± 12
Heart rate (beats/min)	71 ± 13	70 ± 12	71 ± 13
Men	69 ± 13	69 ± 12	69 ± 12
Women	70 ± 12	72 ± 13	71 ± 12
Right finger temperature at 300 s (°C)	32.1 ± 2.7	32.6 ± 1.5	32.2 ± 1.9
Left finger temperature at 300 s (°C)	31.9 ± 2.8	32.6 ± 1.6	32.7 ± 2.0
Room temperature (°C)	24.2 ± 1.7	24.6 ± 2.2	24.4 ± 1.8

**Table 2:** Linear regression model showing associations with vascular reactivity index (VRI).

VRI (dependent)	Coefficients	*p* value
Intercept	1.88	<0.001
Age	−0.0078	<0.001
Male	−0.086	<0.001
Systolic blood pressure	−0.000079	0.89
Diastolic blood pressure	0.00046	0.63
Heart rate	0.0028	<0.001
Multiple *R* squared	0.053	
*p* value	<0.001	

## Data Availability

The data is available on Endothelix's VENDYS registry server.
